# New Insights on Fatigue Crack Growth of Reinforced Natural Rubber

**DOI:** 10.3390/polym17233200

**Published:** 2025-11-30

**Authors:** William Amoako Kyei-Manu, Lewis B. Tunnicliffe, Charles R. Herd, Keizo Akutagawa, James J. C. Busfield

**Affiliations:** 1 School of Engineering and Materials Science, Queen Mary University of London, London E1 4NS, UK; w.a.kyei-manu@qmul.ac.uk (W.A.K.-M.); k.akutagawa@qmul.ac.uk (K.A.); 2 Birla Carbon, Marietta, GA 30062, USA; lewis.tunnicliffe@adityabirla.com (L.B.T.); charles.herd@adityabirla.com (C.R.H.)

**Keywords:** fatigue crack growth, carbon black, natural rubber, strain-induced crystallisation, hysteresis, tearing energy, crack tip velocity

## Abstract

The fatigue crack growth of natural rubber reinforced with various grades of carbon black has been studied across a wide range of tearing energies. Carbon blacks differ significantly in structure and surface area, influencing the mechanical and dynamic properties of the compounds. High-structure carbon black compounds exhibit an abrupt and significant step change in crack growth rate. Above the point of this step change, the different carbon black compounds have similar crack growth rates. Prior to this step change, high-structure carbon black compounds show a better crack growth resistance of up to two orders of magnitude compared to low-structure carbon black compounds. These step changes are attributed to strain-induced crystallisation effects. Before the step change, high-structure carbon black compounds nucleate and grow enough crystals at the crack tip to suppress crack growth. Above the step change, the crack tip velocity is too rapid for substantial strain-induced crystallisation to occur, as it is a time-dependent phenomenon. The step change aligns with the crack tip velocity where strain-induced crystallisation is reportedly absent. Understanding these step changes in crack growth can help rubber compounders design materials with improved crack growth resistance, leading to more durable components.

## 1. Introduction

In practice, rubber products rarely fail in a single loading cycle. Instead, they typically fail from repeated dynamic loading and unloading, leading to failure from cyclic fatigue crack growth. Understanding the fatigue life of rubber is therefore critical for predicting the failure of rubber materials in various engineering applications. Fatigue life can be defined as the duration or number of loading cycles required for a crack to expand to a critical length, leading to catastrophic mechanical failure or compromising the product’s operational functionality [1,2]. Understanding crack growth is also important for failure modes such as abrasive and fatigue wear in rubber where wear mechanisms occur from slowly propagating cracks [3]. Cut and chip wear can also be driven by crack growth [4].

Mechanical loading of the rubber material, the environmental conditions that the rubber material has been exposed to, the constitutive behaviour of rubber, and constituents of rubber formulation are some of the broad factors that influence the fatigue life of rubber [5]. In strain-crystallising rubbers, having a non-zero minimum strain in dynamic loading significantly reduces crack growth rates [6,7]. Rubbers that strain-crystallise are not significantly affected by the frequency of loading in the range of $10^{-3}$ to 50 Hz [8]. For natural rubber which crystallises, fatigue life decreases by a factor of 4 when temperature increases from 0 °C to 100 °C, while for styrene butadiene rubber which does not crystallise, fatigue life drops by a factor of $10^{4}$ over the same temperature range [9].

The inclusion of reinforcing particulates such as carbon black enhances the fatigue life relative to the unreinforced equivalent [10]. The level of enhancement is dependent on the type [2,10] and loading [2] of the carbon black. Carbon black enhances fatigue life through various mechanisms including enhancing the stiffness and hysteresis of the resulting compound, crack tip blunting, deviation and branching resulting from non-homogeneity of the rubber-particulate composite at the crack tip [5]. Strain amplification at the crack tip from the inclusion of particulates is also reported to promote the formation of strain-induced crystallites ahead of the crack which inhibit crack growth [2].

Despite significant progress in understanding crack growth dynamics, there are aspects of crack growth that are still unknown [5]. This is especially true for reinforced, strain-crystallising rubber formulations because of the constitutive behaviour of these compounds which can produce opposing effects. For example, while particulates generally enhance crack growth resistance, they could also increase the effective initial flaw size which could enhance crack nucleation and propagation [5]—especially when they are not effectively dispersed.

The main objective of this paper is to enhance our understanding of the fatigue life of carbon-black-reinforced natural rubber compounds. Natural rubber reinforced with eight different grades of carbon black, varying in their structure and surface area, is studied over a wide range of tearing energy. A tearing energy range (from ~500 ${Jm}^{-2}$ to ~14,000 ${Jm}^{-2}$ depending on the compound) wider than typically tested and reported in the literature was chosen to potentially capture any interesting aspects of the fatigue crack growth behaviour of the tested compounds.

### Evaluation of Crack Growth Using Fracture Mechanics

Crack growth is evaluated using a fracture mechanics approach originally postulated by Griffith [11] and later modified by Rivlin and Thomas [12] for rubber materials. According to fracture mechanics, for a purely elastic material with no yielding behaviour, a crack will grow if the elastic energy released upon its growth is greater than the surface energy generated upon the creation of two new surfaces when the crack grows. The elastic strain (stored) energy released upon the growth of a crack area, *da*, is referred to as the tearing energy, $T_{E}$, as shown in Equation (1) where *U* is the strain energy in the rubber specimen [11,12].

(1) $$T_{E}=-{\left(\frac{dU}{da}\right)}_{l}$$

**Figure 1:**
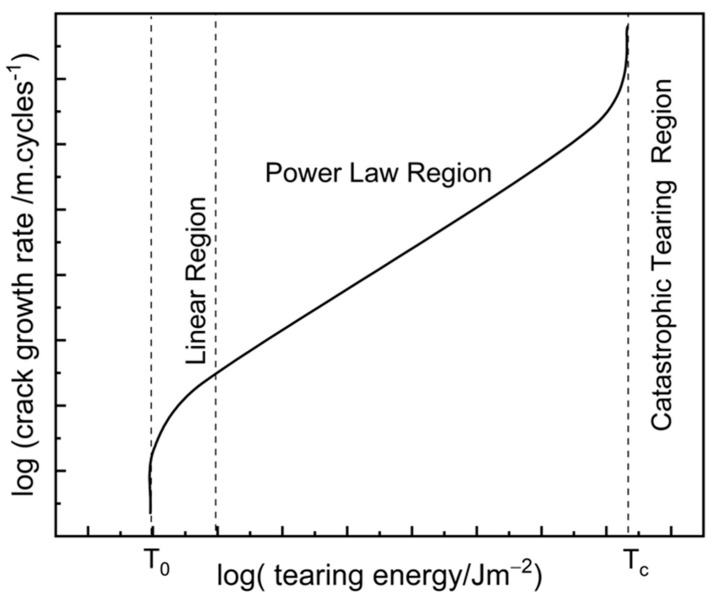
Representation of fatigue crack growth behaviour for rubber. Adapted from Lindley [7].

Tearing energy can be calculated directly from the external forces applied to certain test geometries and is independent of crack length for the case of pure shear/planar tension geometry. Figure 1 shows a schematic of a typical crack growth rate versus tearing energy behaviour of a rubber material.

Below the fatigue threshold/intrinsic strength, $T_{0}$, there is no mechanical crack growth. Crack growth below these tearing energies is chemically driven through ozone scission of polymer chains. The fatigue threshold of rubber materials is typically of the order of 10 to 100 $Jm^{-2}$ [13,14,15,16]. Immediately ahead of the fatigue threshold/intrinsic strength, crack growth is dependent on both ozone and mechanical factors in an approximately additive and linear manner. At a critical tearing energy, $T_{c}$, the failure process transitions to a single cycle of catastrophic tearing. The critical tearing energy is usually around 10 to 100 $kJm^{-2}$ for rubber materials.

Between $T_{0}$ and $T_{c}$ (and immediately ahead of the linear region), the crack growth rate shows a power law relationship with tearing energy as shown in Equation (2), where *c* is the crack length, *n* is the number of fatigue cycles, and A and β are material-dependent parameters. For practical reasons, most experimental crack growth measurements are performed in the power law region.

(2) $$\frac{dc}{dn}=AT_{E}^{\beta }$$

Of scientific and industrial significance are transitions in the crack growth behaviour during testing which were first reported by Kadir and Thomas [17] over 30 years ago. For a non-crystallising styrene butadiene rubber compound, they observed three regions of tearing: a slow crack growth region at low tearing energies, a fast crack growth in high-tearing-energy regions, and a stick slip region which acted as the transition region between these two regions. They attributed the change in crack growth rates to competing effects of cavitation occurring at low tearing rates and an increase in modulus (termed glassy modulus) at high tearing rates of rubber.

Researchers including Busfield et al. [18] have also observed an abrupt transition in crack growth behaviour. They quantified the time-dependent and cyclic components influencing crack growth in styrene butadiene rubber, using crack growth constants from static measurements at constant tearing energy and assessed how frequency affects cyclic crack growth. The cyclic crack growth behaviour was dominated by time-dependent crack growth at low frequency and high tearing energy, while high frequency and low tearing energy favoured cyclic crack growth. This transition in crack growth behaviour was attributed to the formation/melting of quasi-crystals at the crack tip.

Kubo et al. [19] also observed an abrupt change in crack propagation velocity by typically more than two orders of magnitude at a specific threshold strain energy which they referred to as a “velocity jump”. Using theoretical analysis, numerical calculations, and experiments using unfilled acrylonitrile butadiene rubber, styrene butadiene rubber, and butadiene rubber, they argued that the velocity jump was due to dynamic glass transition at the crack tip. They suggested that the velocity jump could be found in general viscoelastic materials.

A detailed inspection of the fatigue crack growth curves of the tested compounds is performed to determine if these abrupt transitions are observed in the reinforced natural rubber compounds.

## 2. Materials and Methods

### 2.1. Materials

**Figure 2:**
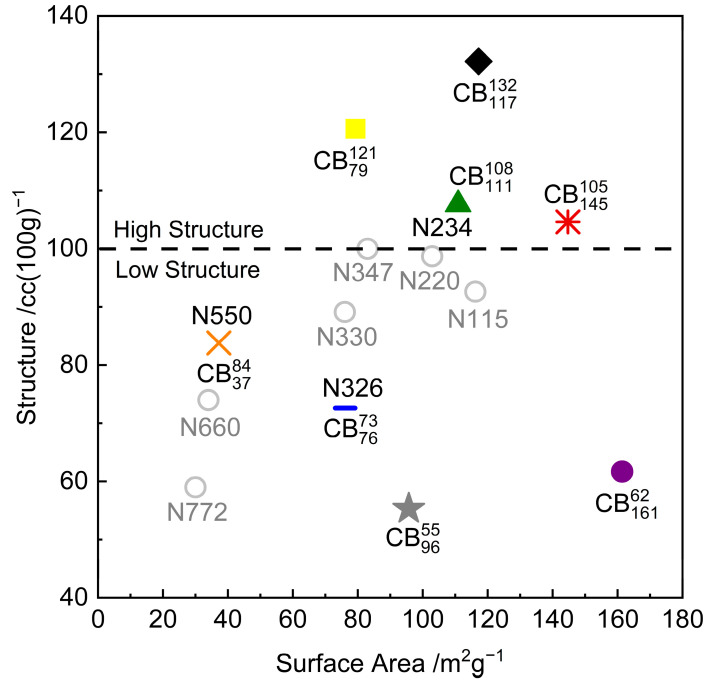
Colloidal plot of tested and reference carbon black grades. The dash line at 100 $cc\left(100\right)g^{-1}$ is used to differentiate between carbon black grades that are referred to as high structure and those referred to as low structure in this paper.

Eight different compounds of SMR CV60 natural rubber were tested, each reinforced with a different grade of carbon black at 50 parts per hundred (phr) loading. Table S1 in the Supplementary Materials shows the formulation of the compounds. The carbon blacks were selected to cover a wide range of surface areas and structures as seen in Figure 2. The eight carbon blacks tested in this study are shown as coloured symbols. The grey open circles represent some common ASTM carbon black grades. These are not tested in this study and are only included in Figure 2 to contextualise the surface area and structure of the tested carbon black grades. An unfilled equivalent is also included for reference.

In this study, the terms *high-structure carbon blacks*/*compounds* and *low-structure carbon blacks*/*compounds* are often used. These terms are used to differentiate between the four carbon blacks that have a structural value (as measured by the compressed oil absorption number (COAN) following ASTM D3493 [20]) above 100 $cc\left(100\right)g^{-1}$ and the four carbon blacks that have a structural value below 100 $cc\left(100\right)g^{-1}$. The structural value of 100 $cc\left(100\right)g^{-1}$ is arbitrarily chosen to delineate the two structural levels due to the differences in the crack growth behaviour of the two sets of compounds.

A naming convention that immediately identifies the structure and surface area of the carbon black was adopted for this study. The carbon black structure measured by the compressed oil absorption number (COAN) is listed as the superscript, while the carbon black surface area measured by the statistical thickness surface area (STSA) following ASTM D6556 [21] is listed as the subscript. For example, N550 carbon black is referred to as ${CB}_{37}^{84}$ since it has COAN and STSA values of 84 $cc\left(100\right)g^{-1}$ and 76 $m^{2}g^{-1}$, respectively. Table 1 shows the carbon black structure and surface area, as well as the adopted naming convention.

The compounds were mixed in a 1.6 L Banbury mixer at Birla Carbon, Marietta, GA, USA. The compounds were mixed in a three-stage mixing process (as detailed in Supplementary Tables S2–S4) to ensure full macro-incorporation of the carbon black in the compounds. Table 1 shows the interferometric microscope (IFM) dispersion index (DI) values of the final compounds measured according to ASTM D2663 (method D) [22]. The compounds show high DI values indicating full macro-incorporation in line with the dispersion indices observed for commercially prepared rubber compounds.

### 2.2. Crack Growth Measurements

A pure shear fatigue crack growth specimen [23,24] was used for the fatigue crack growth measurements of the compounds. The original width and thickness of the specimen was ~155 mm and ~2 mm, respectively. The initial length of the specimen as measured by the distance between the upper and lower clamp was ~13 mm.

An initial cut of ~20 mm was made in the specimen using a sharp blade and the specimen was cycled at a crosshead displacement from 0 mm to 1.75 mm for 5000 cycles before commencing the measurements. The preconditioning cycling of the crack tip was performed to minimise any artificial sharpness of the crack profile resulting from the cutting blade which could affect the initial crack growth rate before steady state crack growth measurements were made [25].

The samples were cycled at specific strains for a defined number of cycles as shown in Table 2. The frequency for the first four displacement levels (2.00 mm, 2.25 mm, 2.50 mm, and 3.00 mm) was 4 Hz. The low-structure carbon black compounds were cycled for a higher number of cycles as indicated in the brackets in Table 2 to obtain measurable crack lengths at the lower displacement level. A value of 4 Hz was chosen as the frequency level since it was high enough to minimise the required test time for the significant number of cycles needed at low displacement levels, while also being below the frequency threshold where frequency is reported to influence crack growth measurements [8].

The frequency was 2.5 Hz for the subsequent displacement levels (>3.00 mm) and all of the compounds were cycled to the same total number of cycles. The number of cycles was chosen to obtain a measurable amount of crack growth at each displacement level while also ensuring that the width-to-height aspect ratio stayed at >7 at the end of the test to ensure the specimen was in pure shear for the total duration of the experiment.

The crack growth tests were performed at room temperature using an MTS 831.10 elastomer test system. A sinusoidal fatigue displacement was applied to the sample with a fully relaxing strain condition (*R* ratio = 0). Two repeats were performed for each sample. The temperature during testing was approximately 23 °C ± 3 °C with a relative humidity of ≤55%.

### 2.3. Evaluation Procedure of Crack Growth Measurements

#### 2.3.1. Calculating Tearing Energy

The tearing energy, $T_{E}$, for pure shear specimens is shown in Equation (3) based on the energy balance approach by Rivlin and Thomas [12]. $W^{\prime }$ refers to the strain energy density and $l_{0}$ refers to the initial specimen height.

(3) $$T_{E}=-{\left(\frac{{dU}^{\prime }}{da}\right)}_{l}=W^{\prime }l_{0}$$

From the experimental data, the tearing energy, $T_{E}$, is calculated using Equation (4).

(4) $$T_{E}=\frac{U^{\prime }}{t_{0}(w-c+e)}$$

$U^{\prime }$ is the strain energy which is calculated by integrating the mechanical force–displacement curves, $t_{0}$ is the unstrained specimen thickness, w is the initial specimen width, and c is the crack length. Equation (4) compensates for the reduction in the specimen cross-sectional area due to the progressive crack growth and for the non-zero strain energy of a volume of material in the crack part of the specimen [26]. e is a constant that is used to account for the non-zero strain energy. The value of e is 0.28 $l_{0}$ based on research by Busfield et al. [26].

The tearing energy can be calculated on either the loading or unloading curves. For this paper, the tearing energy was calculated using the unloading curve, which is the norm for most published fatigue crack growth studies [2] because this is the elastic energy released during crack growth [8].

#### 2.3.2. Calculating Crack Growth Rate

At various intervals during testing, the cyclic procedure was paused, and the specimen was strained to the peak displacement of the sinusoidal cycle to fully open the crack. A length-calibrated image of the crack was captured using a Canon EOS Rebel T6 camera (Canon, Tokyo, Japan) equipped with a macro lens. The images were taken at intervals of a 5th of the total number of cycles at the given displacement or strain level. For example, for a total of 100,000 cycles, images were taken on cycles 20,000, 40,000, 60,000, 80,000, and 100,000. An image was also taken at the start of each displacement cycle; thus, a total of six images were taken at each strain level. The total number of cycles was chosen to ensure that a total crack growth of at least 0.5 mm was achieved at each strain level.

The crack length was measured from the images using Image J software(Version 1.54a). The crack growth rate was obtained from the gradient of a best fit line through the measured crack lengths at each specific displacement/strain level. Figure S1 in the Supplementary Materials shows the crack length as a function of the number of cycles for the ${CB}_{117}^{132}$ compound accompanied by images at specific cycles across 3 displacement levels.

The strain, stress, and mechanical hysteresis are calculated as detailed in Section S4 of the Supplementary Materials.

## 3. Results

### 3.1. Stress–Strain Curves, Peak Stress, and Mechanical Hysteresis

**Figure 3:**
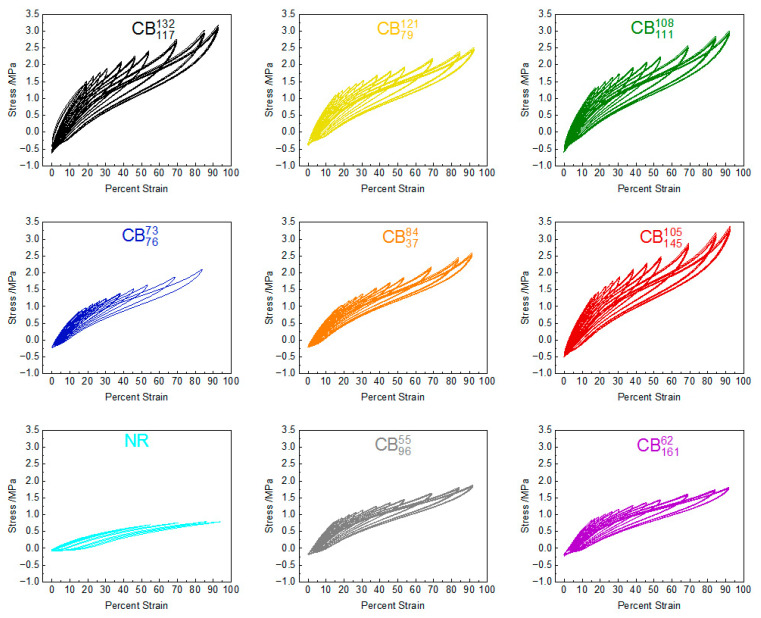
Stress–strain curves during fatigue testing of carbon-black-reinforced natural rubber compounds and unfilled equivalent.

Figure 3 shows the stress–strain curves of the tested compounds during the fatigue crack growth experiments. The graphs only show the stress–strain curves from the cycles when images were taken for crack measurements. These cycles were also used for fatigue crack growth calculations. The graphs generally show testing from ~15% strain to ~92%.

**Figure 4:**
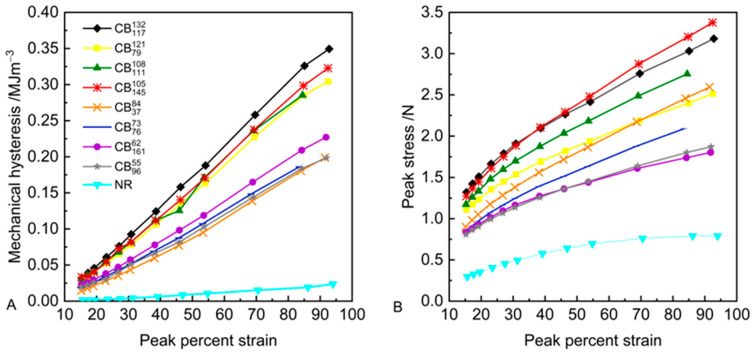
(**A**) Mechanical hysteresis and (**B**) peak stress as a function of peak percent strain during fatigue testing of carbon-black-reinforced natural rubber compounds and unfilled equivalent.

To quantify the mechanical hysteresis of the tested compounds, the mechanical hysteresis on the first recorded cycle is plotted as a function of strain in Figure 4. The peak stress on the first recorded cycle at each strain level is also plotted as a function of strain for the tested compounds.

The mechanical hysteresis increases with increasing strain, as expected, for all of the tested compounds. There are observed differences though in the value of the mechanical hysteresis for the different tested compounds. At equivalent strain levels, the mechanical hysteresis of the high-structure carbon black compounds is higher compared to the low-structure carbon black compounds and the unreinforced equivalent. The differences in the value of the mechanical hysteresis between the high- and low-structure carbon black compounds become more pronounced at medium-to-high strains (≥~20%).

Regression analysis is performed to determine the effect of the carbon black structure and surface area on the mechanical hysteresis and peak stress during fatigue testing of carbon-black-reinforced natural rubber compounds. From the analysis shown in Supplementary Section S5, at equivalent strain levels, both the carbon black structure and surface area influence the calculated mechanical hysteresis, although the carbon black structure has a more significant effect. The peak stress is influenced by the carbon black structure. The carbon black surface area has no statistical influence on the calculated peak stress.

### 3.2. Fatigue Crack Growth Rate

**Figure 5:**
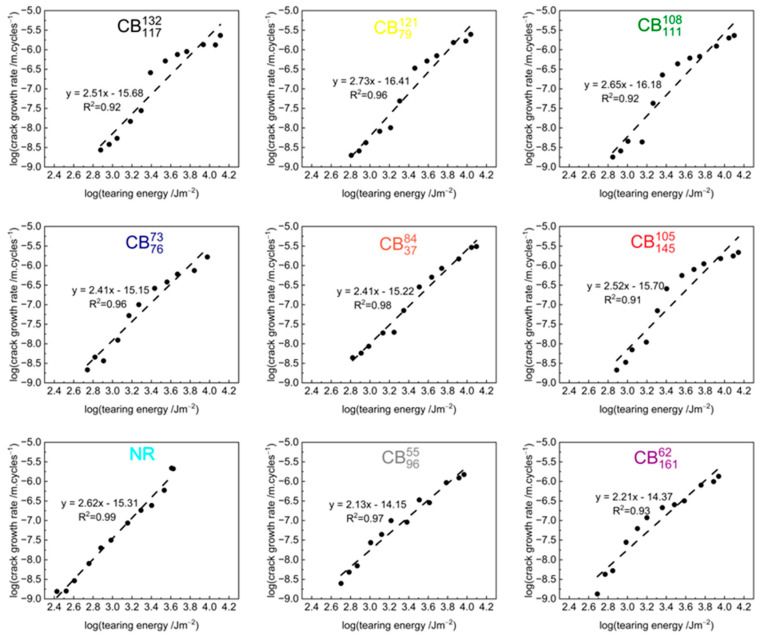
Fatigue crack growth plotted as a function of tearing energy on a log-log scale for carbon-black-reinforced natural rubber compounds and unfilled equivalent.

Figure 5 shows the crack growth rates of the tested compounds as a function of the tearing energy using logarithmic scales on both axes. For each compound, the first tested specimen is shown in the graph.

The fatigue crack growth rate curves show a reasonable correlation with the power law relation in Equation (2). Table 3 summarises the values of the *β*-parameter, which is a characteristic parameter describing how sensitive the crack growth rate is to changes in tearing energy, and the log *A* parameter, which is the intercept of the crack growth rate as a function of the tearing energy graph. For each compound, the average of the two repeats is shown for the *β* and log *A* parameters with the standard deviation shown as the error. The table also shows the $R^{2}$ values of the best fit on the logarithmic scale for each tested specimen of each compound.

The $R^{2}$ values are ≥0.91, suggesting good correlation between the crack growth rate and the tearing energy of the tested compounds. In other words, the crack growth rate and tearing energy of the tested compounds fit the power law relation. The average *β*-parameters range between 2.12 and 2.76 with the average of all of the tested compounds being about 2.46. Typical *β*-parameter values of natural rubber reported in the literature are ~2 [1]. Higher *β*-parameter values (~3–4) have, however, been reported for the single edge notched test (SENT) and a pure shear specimen of natural rubber reinforced with 20, 40, and 60 phr of N234 carbon black and an unfilled equivalent [27]. *β*-parameter values of about 3 and 4 are typically expected for cis-polybutadiene and styrene butadiene rubber compounds [1], but similar values have also been reported in the high-strain regions in the classical literature for natural rubber [1].

The lower *β*-parameter values of natural rubber compounds are generally ascribed to the toughening effects from the formation of crystallites in the highly strained region of rubber directly ahead of the crack tip. The formation of the crystallites increases the mechanical hysteresis and energy dissipation at the crack tip. The crystallites also cause crack tip deflection. Both phenomena contribute additional toughening mechanisms that decrease the crack growth rate [1,2].

It is important to note that stiff compounds cause a sharpening of the crack tip which reduces the volume of highly strained rubber ahead of the crack. This effect could apparently overwhelm the promotion of strain-induced crystallisation at the crack tip [2,28,29,30]. Thus, there may be a more complex interplay between the crack growth behaviour and the compound’s mechanical properties. Specifically for the tested compounds, the structure of the carbon black affects the mechanical properties, with increasing structure leading to higher stiffness as discussed in Section 3.1 and in previous publications [31] that studied the static and dynamic properties of the tested compounds.

**Figure 6:**
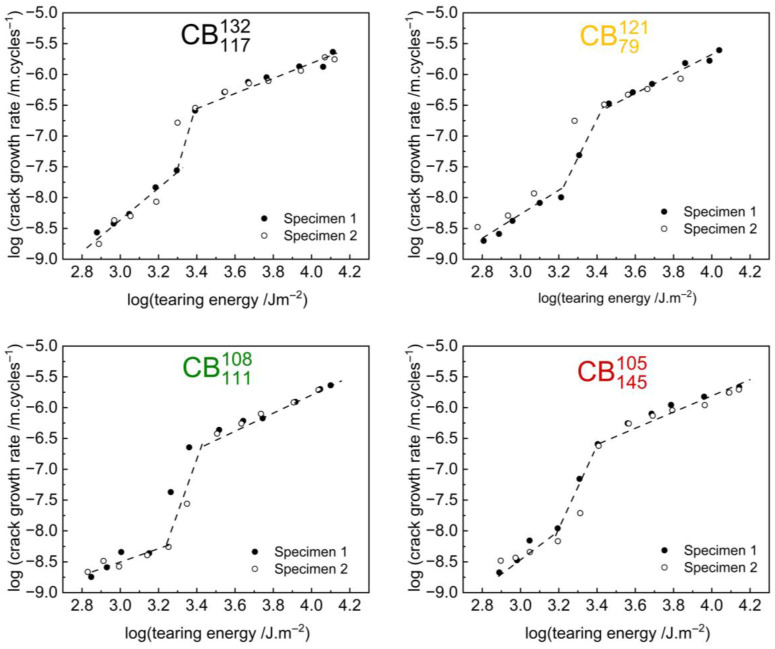
Fatigue crack growth rate plotted as a function of tearing energy on a log-log scale for the high-structure carbon black compounds. Repeats of experiments are shown as open circles and also show the step change. Guiding lines are shown to indicate the transition in crack growth rate.

Indeed, a closer inspection of the crack growth rate curves shows more complex behaviour, especially for the high-structure carbon black compounds. Figure 6 reproduces the crack growth curves of the high-structure carbon black compounds. The repeats of the experiments are superimposed on the graphs as open circles and show that the experiments had good repeatability. Both the initial experiments and the repeats show an abrupt step change at a tearing energy of $T_{E}=\sim 2500\;Jm^{-2}$ which causes a change of 1–2 orders of magnitude in the crack growth behaviour as shown by the dash guidelines.

The step change is also observed when the crack growth rate is calculated using the tearing energy on loading as seen in Figure S3 in the Supplementary Materials. The repeatability of the observed step change on the loading curves suggests the step changes are not merely due to experimental artefacts. It is worth pointing out though that further analysis as detailed later in this manuscript and in Section S10 of the Supplementary Materials is required to determine if the occurrence of the step specifically at TE ≈ 2500 Jm^−2^ is a material property or a result of the protocol parameters.

Understanding what could cause a change of 1–2 orders of magnitude in the crack growth rate could have significant implications for the design of rubber materials which undergo fatigue failure. A change in crack growth rate from ≈10^−8^ m/cycle to ≈10^−6^ m/cycle significantly impacts the useful lifetime of a rubber product. For example, while 100,000 cycles are needed to grow a crack by 1 mm at a rate of ≈10^−8^ m/cycle, only 1000 cycles are needed to grow a crack by 1 mm at a rate of ≈10^−6^ m/cycle. This difference in crack growth rates could considerably change the useful lifetime of a rubber product.

**Figure 7:**
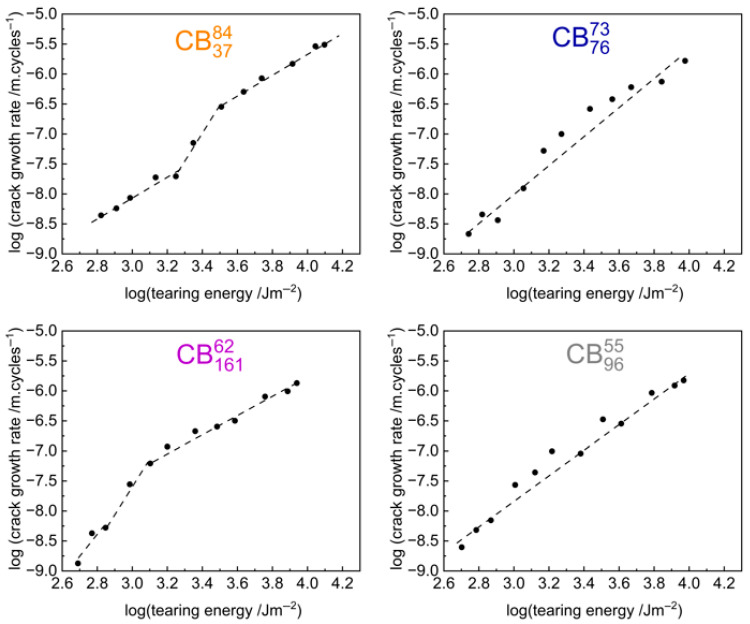
Fatigue crack growth rate plotted as a function of tearing energy on a log-log scale for the low-structure carbon black compounds. The ${CB}_{37}^{84}$ and ${CB}_{161}^{62}$ carbon black compounds also potentially show a step change like that observed for the high-structure carbon black compounds. Guiding lines are shown to highlight the differences in crack growth behaviour.

Figure 7 shows the crack growth curves of the low-structure carbon black compounds. From the graphs, the ${CB}_{37}^{84}$ carbon black compound (which has the highest structure among the four *low-structure carbon black* compounds) seems to show a similar step change at a tearing energy of $T_{E}=\sim 2500\;Jm^{-2}$. The ${CB}_{161}^{62}$ carbon black compound (which has the highest surface area among the tested carbon black compounds) also seems to show a step change—albeit it to a smaller extent and at a much lower tearing energy value of $T_{E}=\sim 1000\;Jm^{-2}$. Table S5 in the Supplementary Materials lists the tearing energy values immediately prior to and after the step change for the high-structure carbon black compounds and the ${CB}_{37}^{84}$ and ${CB}_{161}^{62}$ compounds.

## 4. Discussion

### 4.1. Possible Explanations of Step Change

The exact reason for the observed step change in the crack growth rate remains unknown. Table 4 summarises two possible reasons that could be proposed to explain the observed step change and why they may not apply in this case. A detailed discussion of both reasons is provided in Section S7 of the Supplementary Materials.

### 4.2. Most Likely Explanation of Step Change: Strain-Induced Crystallisation Effects

**Figure 8:**
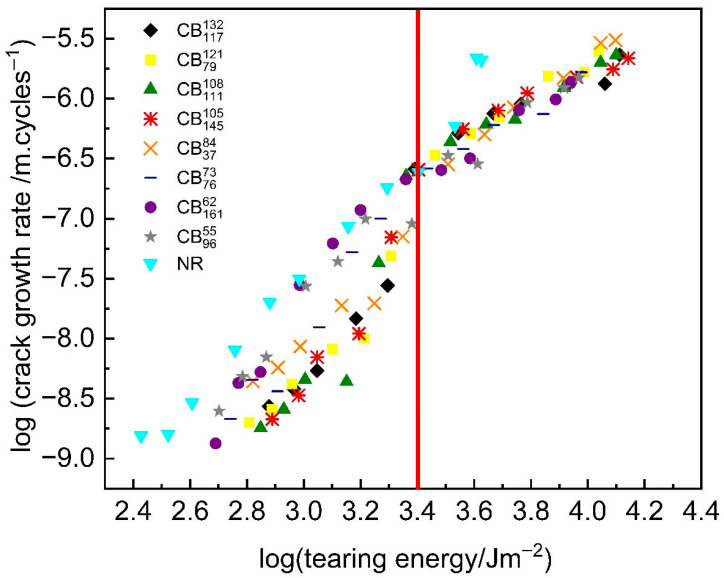
Fatigue crack growth rate plotted as a function of tearing energy for all tested compounds on the same log-log scale axis. The red line shows the tearing energy ($T_{E}=\sim 2500\;Jm^{-2}$) where the step change occurs, and significant differences in the crack growth behaviour of the tested carbon black compounds are observed.

In the opinion of the authors, strain-induced crystallisation effect is the most likely hypothesis for the observed step change in the crack growth behaviour. Figure 8 shows a plot of crack growth rate as a function of tearing energy for all of the tested compounds on the same axes. A red line is drawn at a tearing energy of ~2500 $Jm^{-2}$. At tearing energies above 2500 $Jm^{-2}$, the crack growth rates of the tested carbon black compounds converge, with very little separation amongst the different carbon black compounds. At tearing energies below 2500 $Jm^{-2}$, however, there is significant separation between the crack growth rates of the tested carbon black compounds.

By plotting the crack growth rates of the tested carbon black compounds on the same axes as shown in Figure 8, the transition can be more specifically described as a *step down* and not just a *step change*. Up until $T_{E}=\sim 2500\;Jm^{-2}$, the high-structure carbon black compounds exhibit better crack growth resistance compared to the low-structure carbon black compounds. Above $T_{E}=\sim 2500\;Jm^{-2}$, however, there is a transition, and there is very little distinction in the crack growth resistance of the tested carbon black compounds.

Chanda et al. [38] made a similar observation in their fatigue crack tests of natural rubber compounds reinforced with carbon blacks of varying particle size and structure. They observed that “*below 25% strain, the crack growth rate decreases with filler structure whereas beyond 25%, all the values were quite similar for all the compounds especially for 70 °C and 100 °C.*”

Enhancement in the crack growth resistance of natural rubber compounds due to the formation of crystals at the crack tip is widely reported and discussed [1,2,6,7,39,40,41,42,43,44,45,46]. Less widely discussed though is the possibility of overcoming the strain-induced crystallisation effect in natural rubber or not forming enough crystals to significantly influence the crack growth behaviour. Salkulkaew et al. [47] investigated the effect of the rate of strain on tearing in rubber and discovered that at fast rates, the kinetics of crystallisation were overcome for some of the tested materials.

The formation of crystals under strain is a time-dependent phenomenon [40,48,49,50] and requires a characteristic relaxation time for the polymer chains to align sufficiently to nucleate the crystals. The time-dependence of strain-induced crystallisation effects is especially critical under dynamic loading conditions, notably at frequencies above 2 Hz where crystallisation is dependent on frequency [40]. The onset of strain-induced crystallisation under dynamic loading conditions is shifted to greater strains compared to quasi-static loading conditions [51]. Previous publications [52,53] have shown that the global strain for the onset of crystallisation under quasistatic conditions for the tested compounds was ~55% to ~130%, with the high-structure carbon black compounds generally having a lower onset global strain.

In Figure 4A, graphs of mechanical hysteresis as a function of strain do not show a sharp increase/upturn in mechanical hysteresis with increasing strain, typically observed and attributed to the onset of crystallisation at medium–high strains in cyclic loading [31]. This hints at the possibility of reduced or maybe even absent considerable formation of crystallites within the tested dynamic strain regions.

The level of crystallinity is known to be affected by dynamic loading conditions, with the degree of crystallinity considerably lower than under similar quasistatic loading conditions [40]. Brüning et al. [54] tested a natural rubber compound reinforced with 40 phr of carbon black at 1 Hz and a maximum strain of 70%. The maximum crystallinity at the crack tip during dynamic loading was only about 1% and the crystalline zone extended only about 100 μm into the sample. The recorded maximum crystallinity of 1% was about half of the crystallinity measured under quasistatic conditions at the same applied global strain of 70%.

Le Cam et al. [55] recently estimated the crystallinity at the crack tip of natural rubber. Crystallinity values of only about 1.5% and 4% at stretch (strain) values of 2.54 (154%) and 1.77 (77%), respectively, were measured. Interestingly, at the maximum strain of 154%, the maximum crystallinity was not measured directly at the crack tip but to the side of it. This was attributed to the “frustration” of SIC due to other mechanisms.

Brüning et al. [54] posed questions regarding the reinforcing effects of the crystallites, if they are formed, ahead of a crack tip: “*Is a degree of crystallinity of 1% significant to enhance crack growth resistance?*” Brüning et al. [54] also queried the effect of the crystallite size: “*Considering the crystallites have a size of 10 nm × 10 nm × 10 nm, the number density of crystallites is roughly two orders of magnitude lower than the density of the chemical crosslinks already present in the material suggesting a negligible effect*.” Young and Danik [56] also observed from fatigue and fracture studies of various rubber compounds that “*when the strain rate was rapid enough, the crack growth occurs too quickly for strain crystallisation to impede it*.”

Based on the above discussion, the following mechanisms are proposed to explain the *step down* observed for the crack growth results of the high-structure carbon black compounds.

1. Above the tearing energy, $T_{E}=\sim 2500\;Jm^{-2}$, the crack growth rate curves converge because the crack tip velocity exceeds the characteristic time needed for strain-induced crystallisation. Consequently, strain-induced crystallisation is suppressed and does not affect crack growth. The tested carbon black compounds, therefore, exhibit similar crack growth resistance, potentially influenced by mechanisms like hysteresis and crack tip blunting from the inclusion of carbon black.
2. Below the tearing energy, $T_{E}=\sim 2500\;Jm^{-2}$, the distinction in the crack growth rates among the different carbon black compounds could be attributed to strain-induced crystallisation effects. Below the tearing energy, $T_{E}=\sim 2500\;Jm^{-2}$, the crack tip velocity is slow enough that the high-structure carbon black compounds form crystals to a more appreciable level to enhance crack growth resistance compared to the low-structure carbon black compounds. This explains the slower crack growth rate observed for these compounds at the low tearing energy and the observed *step down*. Previous publications [52,53] have shown that under quasistatic conditions, high-structure carbon black compounds have an earlier onset of crystallisation and achieve higher levels of crystallinity at similar strain levels compared to low-structure carbon black compounds.

#### Evidence to Support the Strain-Induced Crystallisation Effect Hypothesis

This hypothesis is coincidentally validated in the literature based on a theory proposed by Persson et al. [3]. Persson et al. [3] discuss the need for a characteristic (relaxation) time for strain-induced crystallisation and estimate this characteristic time, *τ*, as *τ* ≈ 0.1 s. Based on Persson et al.’s [3] theory, as the crack tip velocity, *ν*, increases, the amount of strain-induced crystallinity at the crack tip decreases. If the characteristic time for stretching a polymer chain at the crack tip, $t^{*}$, is *shorter* than *τ*, no strain-induced crystallisation occurs at the crack tip. Persson et al. [3] estimated the characteristic time for stretching a polymer chain at the crack tip as

(5) $$t^{*}=\frac{a}{v}$$

where a is the crack tip radius. For a slow-moving crack, Persson et al. [3] estimated the crack tip radius a as 100 nm or less. They therefore estimated and proposed that strain-induced crystallisation may be totally removed for crack tip velocities, v*:*

(6) $$v\approx \frac{a}{\tau }\approx 1\;\mu ms^{-1}$$

To test the proposed hypothesis using Persson et al.’s estimate [3], the crack growth data is replotted as crack growth over time ($\frac{dc}{dt}$) instead of crack growth over the number of cycles ($\frac{dc}{dn}$) to obtain a crack growth velocity as a function of tearing energy.

**Figure 9:**
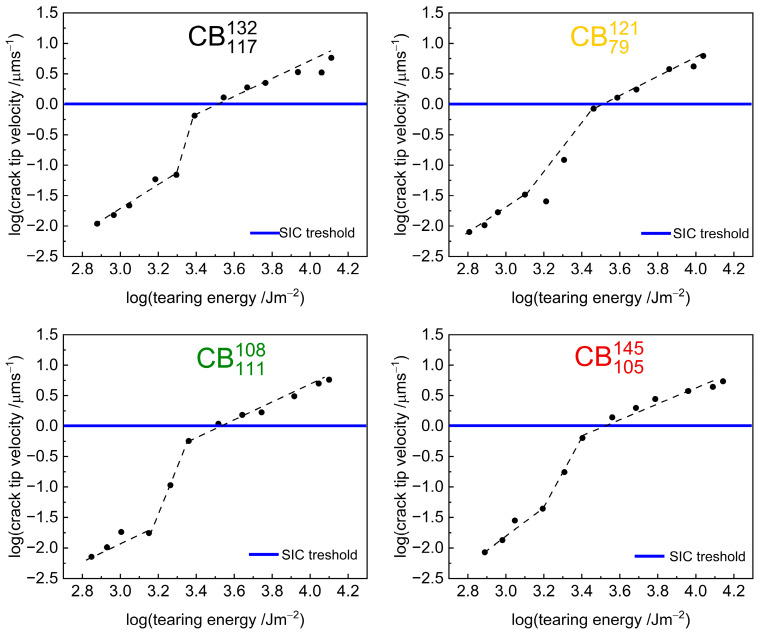
Crack tip velocity plotted as a function of tearing energy on a log-log scale for high-structure carbon black compounds. The blue lines represent a crack tip velocity where Persson et al. [3] predict strain crystallisation may be totally removed from the crack tip.

Figure 9 shows a plot of the crack tip velocity as a function of tearing energy on a log-log scale for the high-structure carbon black compounds. Superimposed on the graphs is a solid blue line showing a crack tip velocity of $1\;\mu ms^{-1}$. This line is labelled as the “SIC threshold”, indicating the crack tip velocity above which Persson et al. [3] predict that strain-induced crystallisation may be overcome. The point at which the crack growth rate begins to *step down* matches with this estimated strain-induced crystallisation threshold of the crack tip velocity. A similar plot is shown in Figure S4 in the Supplementary Materials for the low-structure carbon black compounds. Unlike the high-structure carbon black compounds which show a *step down*, the low-structure carbon black compounds do not show a similar step down at the crack tip velocity where it is predicted that strain-induced crystallisation is overcome.

This helps to validate the argument that at the point of the *step down*, the compounds reinforced with high-structure carbon blacks (which generally have higher levels of crystallinity at similar strain levels and an earlier onset of crystallinity compared to low-structure carbon black compounds due to strain amplification effects [52,53]) may be able to nucleate enough crystals at the crack tip to cause an orders-of-magnitude reduction in crack growth rate.

If confirmed, these observations could have a significant impact on the design of rubber products, especially those such as tire liners where stiff rubber products are most often used. The rubber compounder could adjust formulations to leverage this threshold where the *step down* in crack growth rate occurs to prolong the lifetime of rubber compounds. Further experiments as detailed in Supplementary Section S10 where the effects of crystallisation are either enhanced, minimised, or eliminated can help to validate the proposed hypothesis. Additionally, experiments where the crystallinity at the crack tip is estimated during fatigue crack growth testing can be leveraged. Different crack growth methodologies can also be used to validate that the observed step change is a material property rather than a function of test parameters.

Based on the observed step changes in the crack growth behaviour and the proposed reasons for the change, the following mechanisms that influence the crack growth behaviour at various tearing energy/strain levels are proposed (Table 5).

## 5. Conclusions

The fatigue crack growth of natural rubber compounds reinforced with various grades of carbon black differing in structure and surface area was studied. A significant step down was observed in the fatigue crack growth of high-structure carbon black compounds. At low tearing energies, these compounds show a better crack growth resistance by 1–2 orders of magnitude. The enhancement in crack growth resistance is primarily attributed to strain-induced crystallisation coupled with other effects such as hysteresis resulting from the inclusion of reinforcing fillers such as carbon black. Before the step change, crystallisation inhibits crack propagation. However, beyond a certain strain/tearing energy level, the crack tip velocity becomes too rapid, preventing polymer chains at the crack tip from relaxing and forming crystals. This leads to similar crack growth resistance across all of the carbon-black-reinforced compounds.

This hypothesis is supported by the literature, which suggests the absence of strain-induced crystallisation at specific crack tip velocities. The start of the step down coincides with the proposed crack tip velocities in the literature where it is reported that strain crystallisation may be absent.

Understanding this step down could significantly impact rubber material design once validated through further testing using non-crystallising rubber materials and different testing methodologies. Validating the source of the step change could provide deeper insights into the roles of crystallisation and hysteresis during crack propagation, enabling rubber compounders to design products with improved crack growth resistance and extended lifetimes.

## Figures and Tables

**Table 1:** Carbon black structure and surface area values, adopted compound-naming conventions, and compound dispersion index.

ASTM Grade Name	Carbon Black/Adopted Compound Name	$\mathrm{Structure}\;(\mathrm{COAN})/cc{\left(100g\right)}^{-1}$	$\mathrm{Surface}\;\mathrm{Area}\;(\mathrm{STSA})/m^{2}g^{-1}$	Compound Dispersion Index
NA	${CB}_{117}^{132}$	132	117	99.3
NA	${CB}_{79}^{121}$	121	79	98.8
N234	${CB}_{111}^{108}$	108	111	99.4
NA	${CB}_{145}^{105}$	105	145	98.8
N550	${CB}_{37}^{84}$	84	37	98.7
N326	${CB}_{76}^{73}$	73	76	98.0
NA	${CB}_{161}^{62}$	62	161	81.5
NA	${CB}_{96}^{55}$	55	96	90.2
NA	Unfilled NR	NA	NA	NA

NA = Not Applicable; NR = Natural Rubber.

**Table 2:** Test parameters of fatigue crack growth experiments.

Target Crosshead Displacement/mm	Estimated Percentage Strain Level	Total Number of Cycles/1000 Cycles	Test Frequency/Hz
2.00	15	200 (400)	4
2.25	17	200 (400)	4
2.50	19	150 (300)	4
3.00	23	75 (150)	4
3.50	27	50	2.5
4.00	31	30	2.5
5.00	38	15	2.5
6.00	46	10	2.5
7.00	54	7.5	2.5
9.00	69	2.5	2.5
11.00	85	2.5	2.5
12.00	92	2.5	2.5

**Table 3:** Crack growth power law parameters and the value of best fit of the power law relation.

Tested Compound	Average *β*-Parameter	Average log *A* Parameter	$R^{2}$	
			Specimen 1	Specimen 2
${CB}_{117}^{132}$	2.50 ± 0.01	−15.64 ± 0.06	0.92	0.91
${CB}_{79}^{121}$	2.66 ± 0.11	−16.02 ± 0.55	0.96	0.93
${CB}_{111}^{108}$	2.76 ± 0.16	−16.61 ± 0.61	0.92	0.92
${CB}_{145}^{105}$	2.52 ± 0.00	−15.75 ± 0.07	0.91	0.91
${CB}_{37}^{84}$	2.38 ± 0.04	−15.70 ± 0.68	0.98	0.97
${CB}_{76}^{73}$	2.40 ± 0.02	−15.30 ± 0.21	0.96	0.97
${CB}_{161}^{62}$	2.20 ± 0.02	−14.35 ± 0.03	0.93	0.92
${CB}_{96}^{55}$	2.12 ± 0.01	−14.18 ± 0.04	0.97	0.97
Unfilled NR	2.62 ± 0.00	−15.31 + 0.01	0.99	0.99
Average	2.46	−15.43	NA	NA

**Table 4:** Possible explanations of the observed step change and why they may not apply.

Phenomena	Explanation of Phenomena in Relation to FCG	Possible Explanation for Observed Step Change	Rebuttal for Why the Phenomena Does Not Explain the Observed Step Change
Cavitation	- Defects in the rubber matrix grow into cavities at certain stress concentrations and coalesce behind the crack tip. - A membrane separates the crack tip and cavities. - During crack growth, the membrane tears, the crack tip breaks, and the crack grows into the cavities [32,33,34].	- The high-structure carbon black compounds have higher matrix stress values compared to low-structure carbon black compounds at equivalent strain levels. - At the point of the step change, it could be argued that due to strain amplification effects, the high-structure carbon black compounds exceed a threshold stress for increased cavity formation.	- If a threshold global stress level for enhanced cavity formation exists, the other carbon black compounds should show a similar step change. - The step change occurs at ~31% strain in the high-structure carbon black compounds with an equivalent peak stress range of 1.5 N–1.9 N. - The other carbon black compounds eventually achieve this peak stress but do not show the step change.
Mechanical Hysteresis and Crack Tip Heating	- Intrinsic viscoelasticity leads to hysteresis and temperature increase at the crack tip during cyclic deformation [35]. - Heat evolution diminishes rubber properties and enhances crack growth [36].	- High-structure carbon black compounds show higher hysteresis at equivalent strain levels. - Higher hysteresis at the crack tip could possibly contribute to enhanced degradation leading to the step change in the crack growth rate. - For example, Yamabe et al. [37] recently published fatigue crack growth results of carbon-black-reinforced styrene butadiene rubber materials that showed a step change in the high-tearing-energy regime. They attributed the step change to transition of the crack growth mechanism due to flash heating.	- Low-structure carbon black compounds, however, show similar hysteresis values (0.1 MJm^−3^) to high-structure carbon black compounds when the step change occurs. - Increasing temperature has a less pronounced effect on crack growth rates in natural rubber compared to styrene butadiene rubber [1,29,35]. - Measurement of the specimen temperature using a contact thermocouple of a control sample at the displacement level (4.00 mm) where most of the transition occurs only shows a 0.5–0.6 °C increase in temperature.

**Table 5:** Proposed influences on crack growth resistance of carbon-black-reinforced natural rubber compounds based on tearing energy/strain level.

Description	Proposed Crack Growth Enhancement Level
Low (Strain level ≤ ~30%) ($T_{E}$ ≤ 2500 $Jm^{-2}$)	CB reinforcement effects (such as hysteresis, crack tip blunting, etc.) and SIC due to the strain amplification effect of CB
High (Strain level ≥ ~30%) ($T_{E}\;\geq$ 2500 $Jm^{-2}$)	CB reinforcement effects (independent of CB grade) with no SIC effects

## Data Availability

The original contributions presented in this study are included in the article/Supplementary Material. Further inquiries can be directed to the corresponding author.

## Supplementary Materials

The following supporting information can be downloaded at https://www.mdpi.com/article/10.3390/polym17233200/s1, Table S1: Compound formulation; Table S2: Stage 1 compounding of tested compounds; Table S3: Stage 2 compounding of tested compounds; Table S4: Stage 3 compounding of tested compounds; Table S5: Table showing the tearing energy values before and after the step change in the tested compounds that show a transition; Figure S1: Crack length as a function of number of cycles. The crack lengths were measured using Image J and the crack growth rate was determined by calculating the gradient of the best fit line; Figure S2: Normalised regression coefficient of (A) mechanical hysteresis and (B) peak stress as a function of peak percent strain during fatigue testing of carbon-black-reinforced natural rubber. The open boxes indicate there is no statistical influence based on the regression analysis; Figure S3: Fatigue crack growth rate plotted as a function of tearing energy on loading and unloading on a log-log scale for the high-structure carbon black compounds; Figure S4: Crack tip velocity plotted as a function of tearing energy on a log-log scale for low-structure carbon black compounds. Refs. [1,2,3,29,32,33,34,35,36,54,57,58,59,60,61,62,63,64] cited in Supplementary Materials.
