# Supplementary material for: New Insights on Fatigue Crack Growth of Reinforced Natural Rubber
Source: Polymers (Basel). 2025 Nov 30;17(23):3200. doi: 10.3390/polym17233200 (PMC12693904; doi:10.3390/polym17233200)
Supplement: Supplementary file 1 [file polymers-17-03200-s001.zip › polymers-3952390-supplementary.pdf]

New insights on fatigue crack growth of reinforced natural rubber– Supplementary Information

William Amoako Kyei-Manu[1], Lewis B. Tunnicliffe[2], Charles R. Herd[2], Keizo Akutagawa[1], *James J.C. Busfield[1]

1. School of Engineering and Materials Science, Queen Mary University of London, London E1 4NS, United Kingdom
2. Birla Carbon, Marietta, GA 30062, USA

* Correspondence: j.busfield@qmul.ac.uk; Tel.: +44-20-7882-8866

S1. Formulation of Tested Carbon Black Reinforced Compounds

Table S1 shows the formulation of the carbon black reinforced compounds used for fatigue crack growth testing. The formulation and ingredients for the eight different compounds are the same. The carbon black loading in each compound was similar at 50 phr. The only difference between the compounds is the type of carbon black used for each compound. The carbon blacks varied only in their structure and surface area.

Table S1: Compound formulation.

Component	Loading / parts per hundred rubber (phr)
NR-SMR CV-60	100
Carbon Black	50
Zinc Oxide	5
Stearic Acid	3
Anti-ozonant/Antioxidant	3
Micro-wax	2
Sulphur	2.5
TBBS*-75	0.8

* N-Tertiarybutyl-2-benzothiazole sulfonamide, 75% assay.

S2. Mixing Procedure of Tested Carbon Black Compounds

Tables S2 to S4 show the mixing procedure of the tested compounds. The mixing process is a three-stage process to ensure uniform dispersion of the carbon black in the rubber matrix.

Table S2: Stage 1 compounding of tested compounds

Stage 1: Banbury, 40 °C, 77 rpm, 3.0 bar		
Time/seconds	rpm	Operation
-	77	Load polymer
30	77	Ram down mixing
-	77	Load stearic acid, antiozonant, ZnO, TMQ, Micro-wax. Add all CB blended slowly.
15	77	Ram up mixing
60	77	Ram down mixing
-	77	Sweep
90	77	Ram down mixing
-	77	Sweep
90	77	Ram down mixing
-	77	Sweep
90	77	Ram down mixing (150 °C max, reduce RPM as necessary)

Note: Mill 70 °C, 25:21 rpm, Gap 0.055-60". Do not cut weight back. Pass through the mill once. Band and cross-blend 6 times. Band 30 seconds, sheet off and let cool for a minimum of 1 hour.

Table S3: Stage 2 compounding of tested compounds

Stage 2: Banbury, 40 °C, 77 rpm, 3.0 bar		
Time/seconds	rpm	Operation
-	77	Load master batch (MB)
120	77	Ram down mixing
~150	77	Discharge

Note: Mill 70 °C, 25:21 rpm, Gap 0.055-60". Pass through the mill once. Band and cross-blend 6 times. Band 30 seconds, sheet off and let cool for a minimum of 1 hour.

Table S4: Stage 3 compounding of tested compounds

Stage 3: Banbury, 25 °C, 60 rpm, 3.0 bar		
Make sure master batch weight is correct before starting		
Time/seconds	rpm	Operation
-	60	Load half master batch, curatives and then last half of master batch
30	60	Ram down mixing
30	45	Ram down mixing
-	30	Sweep
120	45	Ram down mixing (100 °C max, adjust RPM as necessary)
~240	45	Discharge

Note: Mill 70 °C, 25:21 rpm, Gap 0.055-60". Pass through the mill once. Band and cross-blend 6 times. Pig it 3 times. Band 30 seconds, sheet off and let cool for a minimum of 1 hour before MDR. Let the compound set for a minimum of 1 hour before curing.

S3. Calculating Crack Growth Rate

Figure S1 displays the crack length as a function of number of cycles for the CB_{117}^{132} carbon black compound, accompanied by images at specific cycles across 3 displacement levels. The crack length was measured on these images using Image J software. The crack growth rate was determined by calculating the gradient of a best-fit line through the measured crack lengths at each specific displacement or strain level.

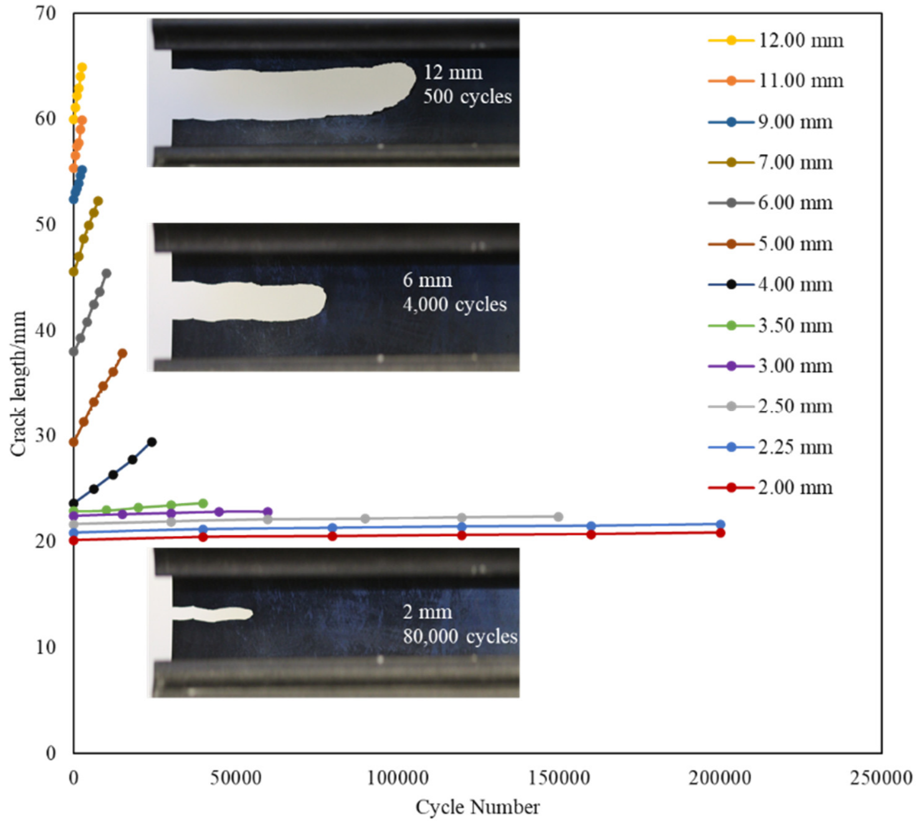

Figure S1: Crack length as a function of number of cycles. The crack lengths were measured using Image J and the crack growth rate was determined by calculating the gradient of the best fit line.

S4. Calculating Strain, Stress and Mechanical Hysteresis

The strain, ε, was calculated as the axial displacement, l, over the initial length, l_0, as measured by the distance between the upper and lower clamp.

$$\varepsilon = \frac{l}{l_0} \times 100 \tag{S1}$$

The stress was calculated using equation S2. Similar to the tearing energy calculation shown in the main manuscript, the stress is calculated to compensate for the reduction in specimen cross-sectional area due to the progressive crack growth.

$$\sigma = \frac{f}{t_0(w-c)} \tag{S2}$$

f is the measured axial force at the point of calculating the stress, t_0 is the unstrained specimen thickness, w is the initial specimen width and c is the crack length at the point of calculating the stress. The peak stress on each cycle is determined from the maximum stress value on each loading cycle.

The mechanical hysteresis is the difference between the strain energy on loading, U_{L} and unloading, U_{UL}.

S5. Regression Analysis of Carbon Black Structure and Surface Area Effects on Mechanical Hysteresis and Peak Stress during Fatigue Crack Growth testing

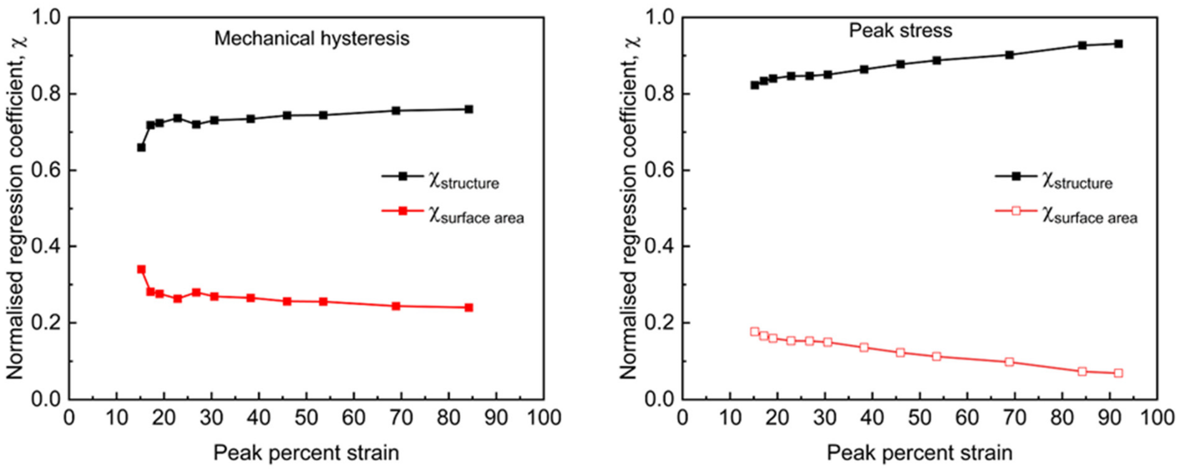

Figure S2: Normalised regression coefficient of (A) mechanical hysteresis and (B) peak stress as a function of peak percent strain during fatigue testing of carbon black reinforced natural rubber. The open boxes indicate there is no statistical influence based on the regression analysis.

Figure S2A shows the normalised coefficients of a multiple regression analysis to determine the effect of carbon black structure and surface area on the calculated mechanical hysteresis at each strain level. At equivalent strain levels, both carbon black structure and surface area influence mechanical hysteresis, although carbon black structure has a more significant effect. Under strain-controlled deformation conditions, high structure carbon blacks enhance the stress level of the compound at a given deformation due to strain amplification effects and therefore increases the absolute amount of energy dissipated [1]. Carbon black surface area enhances mechanical hysteresis due to increased polymer-particulate interaction.

The peak stress shows a similar trend to mechanical hysteresis. The high structure carbon black compounds have higher peak stress values at equivalent strain levels. From figure S2B, carbon black surface area has no statistical influence on the peak stress at equivalent strain levels. These observations can be attributed to strain amplification effects in the rubber matrix due to occlusion

of a certain volume of rubber by the carbon black aggregate structure [2] [3]. Higher structure leads to high strain amplification effects and the observed higher mechanical hysteresis and peak stress.

S6. Fatigue Crack Growth Rates of High Structure Carbon Black Compounds highlighting Step Change on Loading and Unloading cycle.

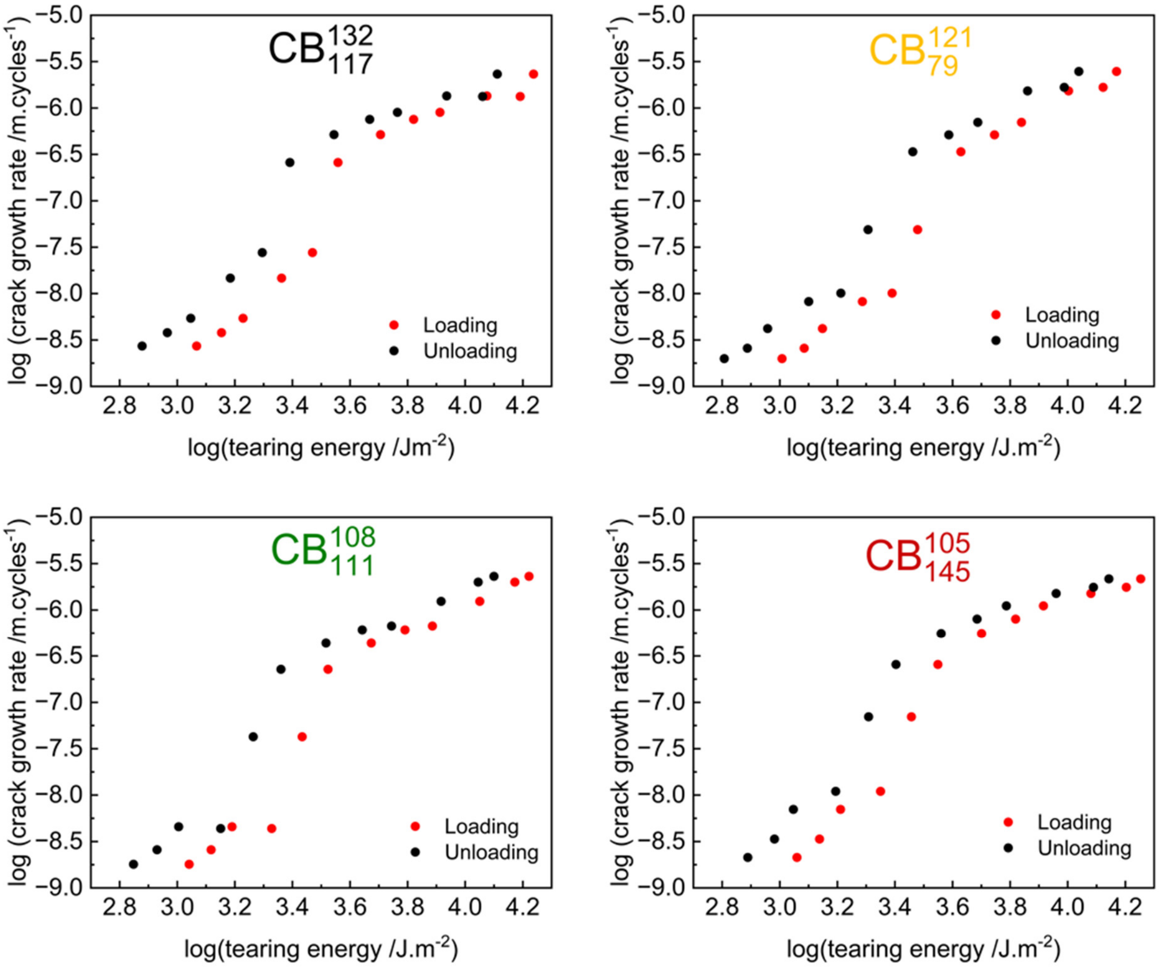

Figure S3: Fatigue crack growth rate plotted as a function of tearing energy on loading and unloading on a log-log scale for the high structure carbon black compounds.

S7. Detailed Analysis of Potential Reasons for Step Change and why they may not apply in this case.

S.7.1 Cavitation

The onset of cavitation could be proposed to explain the observed step change in the crack growth rate behaviour. Cavitation is understood to be a dominant influence in fatigue damage with defects in the rubber matrix growing into cavities and developing into microcracks. The microcracks coalesce behind the crack tip [4] [5] [6].

The exact mechanism of cavitation in rubber is still not fully understood. Some authors attribute it to decohesion of the rubber from the surface of inclusions such as zinc oxide [4] [7] and / or carbon black aggregates [7]. Other authors attribute it to smaller inhomogeneities such as cross-link distribution which act as the nuclei of cavitation and thus cavitation occurs in the polymer matrix rather than at the polymer-particulate / inclusion interface [8]. Regardless of the proposed mechanism, the nucleation and growth of tiny defects into cavities is often attributed to strain amplification occurring from stress concentrations on the mesoscale.

As was shown in figure 4B in the main manuscript, at equivalent strain levels, the high structure carbon black compounds have higher rubber matrix stress values compared to the low structure carbon black compounds. At the point of the step change, it could be purported that due to strain amplification effects, the high structure carbon blacks exceed a threshold stress for cavity and microcrack formation. The crack tip might be tearing into these microcracks formed ahead of it thus causing a more rapid increase in crack length and the concomitant step change in crack growth rate.

The question arises though, assuming there is a threshold global stress level for enhanced cavity formation which eventually leads to the step change in crack growth, why do the other low structure carbon black compounds not show a similar step change even at high tearing energy (or

strain) levels? The global strain level at which the step change occurs for the high structure carbon black compounds is ~31% at which point the peak stress recorded for the high structure carbon black compounds ranges between ~1.5 N to 1.9 N. The low structure carbon black compounds eventually achieve similar peak stress values albeit at higher strain levels but do not show the step change in crack growth rate observed in the high structure carbon blacks.

S.7.2 Mechanical Hysteresis and Heating Effects at Crack Tip

When rubber deforms cyclically, hysteresis occurs. Hysteresis originates from the intrinsic viscoelasticity of the rubber and for rubber compounds such as natural rubber that are amenable to it, from processes such as the formation and melting of crystals during stretching. Hysteresis leads to the evolution of heat. The hysteresis and accompanying heat production could have differing effects on the rubber.

During crack propagation in rubber, heat produced in the vicinity of the crack tip could increase the temperature close to the crack tip [9]. By monitoring the surface temperature during crack growth experiments, Schieppati et al. [10] observed a 5-8 °C increase in temperature at the crack tip. Temperature changes are known to influence crack growth behaviour. Lake and Lindley [11] observed a 4-order of magnitude drop in fatigue life for SBR elastomers going from 0 °C to 100 °C. It is therefore reasonable to assume that the step change in crack growth behaviour could be attributed to heat generation at the crack tip which could contribute to the degradation and fatigue failure of the rubber. This argument could be supported by the observation from figure 4A, that the high structure carbon black compounds have higher mechanical hysteresis compared to the low structure carbon black compounds at equivalent strain levels.

Similar to the counter argument to the previously cavitation theory though, the low structure carbon black compounds eventually show similar hysteresis values (~ $0.1\ \mathrm{MJm^{-3}}$) to those

calculated for the high structure carbon black compounds when the step change occurs. It is also worth highlighting that the effect of increasing temperature in decreasing crack growth rates is less pronounced in natural rubber. For example, while there was a 4-order magnitude drop in fatigue life going from 0 °C to 100 °C in unfilled SBR from Lake and Lindley's experiments, there was only a 4-fold reduction for unfilled natural rubber [11]. The inclusion of particulates even further reduces the temperature dependence of crack growth behaviour [12] [13]. Schieppati et al. [10] concluded from their experiments that temperature increase during cyclic loading depended on testing frequency, with higher frequency causing a greater temperature rise at the crack tip. The frequency of testing, however, had a more significant influence on the measured crack growth rate than the accompanying temperature increase [10]. It is therefore unlikely that the step change in crack growth behaviour could be attributed to degradation of the rubber at the crack tip due to heating effects from hysteresis.

Hysteresis in the rubber also minimises crack growth [14]. Hysteresis reduces the amount of energy input that goes into deforming the polymer chains and propagating cracks [15]. In this regard, local hysteresis in the viscoelastic process zone ahead of the crack tip enhanced by the presence of carbon black requires the input of additional work to drive crack growth versus the corresponding unfilled rubber.

S8. Crack Tip Velocity as a function of Tearing Energy for Low Structure Carbon Black Compounds.

Figure S4 shows the crack tip velocity as a function of tearing energy on a log-log scale for the low structure carbon black compounds. Superimposed on the graphs is a solid blue line showing a crack tip velocity of $1\mu ms^{-1}$. The line is labelled as the *SIC threshold*, indicating the crack tip velocity above which Persson et al. [3] suggest that strain induced crystallization is overcome. Unlike figure 11 in the main manuscript which shows a similar plot for the high structure carbon black compounds and shows a step down that coincides at the indicated crack tip velocity, the low structure carbon black compounds do not show a step down.

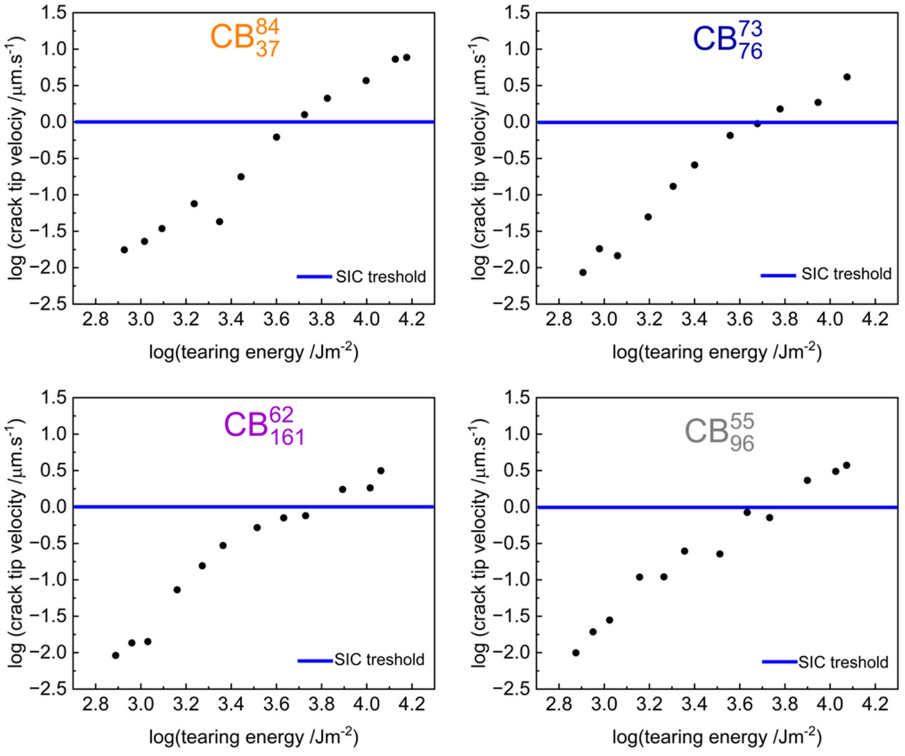

Figure S4: Crack tip velocity plotted as a function of tearing energy on a log-log scale for low structure carbon black compounds. The blue lines represent a crack tip velocity where Persson et al. [9] predict strain crystallisation may be totally removed from the crack tip.

S9. Table showing the tearing energy immediately before and after the step change

Table S5: Table showing the tearing energy values before and after the step change of the tested compounds that show a transition

	Point **before** of step change / $J \cdot m^{-2}$		Point **after** of step change / $J \cdot m^{-2}$	
Tested compound	Sample 1	Sample 2	Sample 1	Sample 2
CB_{117}^{132}	1972	1993	2460	2465
CB_{79}^{121}	1917	1630	2738	2895
CB_{111}^{108}	2051	2033	2559	2533
CB_{145}^{105}	1787	1836	2232	2292
CB_{37}^{84}	1359	1407	1772	1821
CB_{161}^{62}	1073	1447	990	1278

S10. Further Studies to Validate Strain Induced Crystallisation Hypothesis

While the hypothesis of overcoming strain induced crystallisation seems to have some validity based on the literature as shown in the main manuscript, additional experiments need to be performed to further confirm it. Testing with a non-crystallising rubber compound such as styrene butadiene rubber with similar volume fraction and type of carbon blacks under similar test conditions is recommended. The current compound formulation can be adjusted, for example by including higher volume fraction of plasticizers, to observe the changes on strain induced crystallisation of the compounds and the concomitant effect on the observed step change in the crack growth rate. The testing conditions could also be varied to minimise the possible suppression of strain-induced crystallisation. This could include testing at lower frequencies / slow rates (for example below 2 Hz) to minimise the kinetic effects of strain induced crystallisation at the crack tip. Also, instead of a fully relaxing strain condition (R-ratio = 0), a minimum strain can be applied during the cyclic testing. A non-relaxing loading condition has been shown to lead to better crack growth resistance since the crystalline zone continues to exist even at the minimum strain due to the high stress concentration [16]. Although as shown in figure 7 in the main manuscript, repeats of the tests showed a similar *step down* for the high structure carbon black compounds, the experiments could be repeated on a different instrument that can adopt a different protocol. A protocol that could be used is one proposed and previously used by Goosens and Mars [17] where the peak strain is ramped continuously from a minimum starting strain to a large ending strain that successive cycle increases the driving force experienced by the crack. These tests would help validate if the transition observed is indeed a material property and not just a function of the test parameters. A previously published methodology by Le Cam et al. [18] where thermography techniques are used to estimate the strain induced crystallisation at the crack tip could be leveraged

to estimate the crystallinity at the crack tip during crack growth testing. A closer analysis of the crack tip using microscopy techniques could also provide some insight into the development of the crack.

REFERENCES

[1] W. Kyei-Manu, L. Tunnicliffe, C. Herd, K. Akutagawa, O. Kratina, R. Stoček and J. Busfield, "Effect of Carbon Black on Heat Build-up and Energy Dissipation in Rubber Materials," in *Advances in Polymer Science*, Berlin, Heidelberg, Springer, 2024.

[2] M.-J. Wang, S. Wolff and E.-H. Tan, "Filler-elastomer interactions. Part VIII. The role of the distance between filler aggregates in the dynamic properties of filled vulcanizates," *Rubber Chemistry and Technology,* pp. 178-195, 1993.

[3] A. I. Medalia, "Effect of carbon black on dynamic properties of rubber vulcanizates," *Rubber Chemistry and Technology,* vol. 51, no. 3, pp. 437-523, 1978.

[4] J. -B. Le Cam, B. Huneau, E. Verron and L. Gornet, "Mechanism of Fatigue Crack Growth in Carbon Black Filled Natural Rubber," *Macromolecules,* vol. 37, no. 13, pp. 5011-5017, 2004.

[5] T. Glanowski, Y. Marco, V. Le Saux, B. Huneau, C. Champy and P. Charrier, "Fatigue crack initiation around inclusions for a carbon black filled natrual rubber:an analysis based on microtomography," in *Constitutive Models of Rubber XI: Proceedings of the 11th European Conference on Constitutive Models for Rubber (ECCMR 2019)*, CRC Press, 2019.

[6] J. Wu, C. McAuliffe, H. Waisman and G. Deodatis, "Stochastic analysis of polymer composites rupture at large deformations modeled by a phase field method," *Computer Methods in Applied Mechanics and Engineering,* vol. 312, pp. 596-634, 2016.

[7] B. Huneau, I. Masquelier, Y. Marco, V. Le Saux, S. Noizet, C. Schiel and P. Charrier, "Fatigue Crack Initiation in a Carbon Black-Filled Natural Rubber," *Rubber Chemistry and Technology,* vol. 89, no. 1, pp. 126-141, 2016.

[8] E. Euchler , R. Bernhardt, K. Schneider , G. Heinrich, S. Wießner and T. Tada, "In situ dilatometry and X-ray microtomography study on the formation and growth of cavities in unfilled styrene-butadiene-rubber vulcanizates subjected to constrained tensile deformation," *Polymer,* vol. 187, 2020.

[9] B. Persson, O. Albohr, G. Heinrich and H. Ueba, "Crack propagation in rubber-like materials," *Journal of Physics: Condensed Matter,* vol. 17, no. 44, pp. 1072-1100, 2005.

[10] J. Schieppati, B. Schrittesser, A. Wondracek, S. Robin, A. Holzner and G. Pinter, "Impact of temperature on the fatigue and crack growth behavior of rubbers," *Procedia Structural Integrity,* vol. 13, pp. 642-647, 2018.

[11] G. Lake and P. Lindley, "Cut growth and fatigue of rubbers II. Experiments on a noncrystallizing rubber," *Journal of Applied Polymer Science,* vol. 8, pp. 707-721, 1964.

[12] A. Gent, Engineering with Rubber: How to design rubber components, Munich: Hanser, 2012.

[13] A. Thomas, "Rupture of rubber II: The strain concentration at an incision," *Journal of Polymer Science,* vol. 18, no. 88, pp. 177-188, 1955.

[14] L. Tunnicliffe, "Fatigue Crack Growth of Carbon Black-Reinforced Natural Rubber," *Rubber Chemistry and Technology,* vol. 94, no. 3, pp. 494-514, 2021.

[15] J.-B. Le Cam, "Fast Evaluation and Comparison of the Energy Performances of Elastomers from Relative Energy Stored Identification under Mechanical Loadings," *Polymers,* vol. 14, no. 3, p. 412, 2022.

[16] K. Brüning, K. Schneider, S. Roth and G. Heinrich, "Strain-induced crystallization around a crack tip in natural rubber under dynamic load," *Polymer,* vol. 54, pp. 6200-6205, 2013.

[17] J. R. Goossens and W. V. Mars, "Finitely Scoped, High Reliability Fatigue Crack Growth Measurements," *Rubber Chemistry and Technology,* vol. 91, no. 4, pp. 644-650, 2018.

[18] J.-B. Le Cam, W. A. Kyei-Manu, A. Tayeb, P.-A. Albouy and J. J. Busfield, "Strain-induced crystallisation of reinforced elastomers using surface calorimetry," *Polymer Testing,* vol. 131, 2024.
